# Commentary: Impact of the COVID-19 Pandemic on the Mental Health of College Students: A Systematic Review and Meta-Analysis

**DOI:** 10.3389/fpsyg.2021.753798

**Published:** 2021-11-16

**Authors:** Joel Swai, Adam Mohamed, Jing-ping Zhang

**Affiliations:** ^1^Department of Medicine, University of Alberta, Edmonton, AB, Canada; ^2^Department of Internal Medicine, Benjamin Mkapa Hospital, Dodoma, Tanzania; ^3^Mental Health Institute of the Second Xiangya Hospital, Central South University, Changsha, China; ^4^Department of Nursing Management, Xiangya School of Nursing, Central South University, Changsha, China

**Keywords:** college students, coronavirus disease 2019, mental health, depression, anxiety, meta-analysis

## Introduction

Coronavirus Disease 2019 (COVID-19) pandemic has resulted in adverse psychological effects among individuals globally. Recurring infection waves, extended lockdowns, the emergence of more transmissible, and immunity evasive variants are among the unpredictable factors that lead to an increased sense of fear associated with the pandemic (Batista et al., 2021). Moreover, social isolation, quarantines, unemployment, and *long-covid* are strongly associated with depression (Loades et al., 2020; Islam et al., 2021). These issues indicate the need for epidemiological studies exploring the pandemic's effect on mental health.

In a recent meta-analysis study by Li et al. (2021), the authors pooled the prevalence of anxiety and depression associated with COVID-19 among college students. They reported a prevalence of 36% [95% Confidence Interval (CI): 26–46] and 39% [CI: 27–51] for anxiety and depression, respectively, both with statistically significant high heterogeneity of 99.9%, *p* < 0.01. In their study limitations section, authors called upon further heterogeneity analyses by considering the confounding factors. In response, we performed a meta-regression and pooled the prevalence of anxiety and depression stratifying results by potential confounders (i.e., gender, survey instrument used, survey country, and survey dates).

## Prevalence of Anxiety

**Figure 1:**
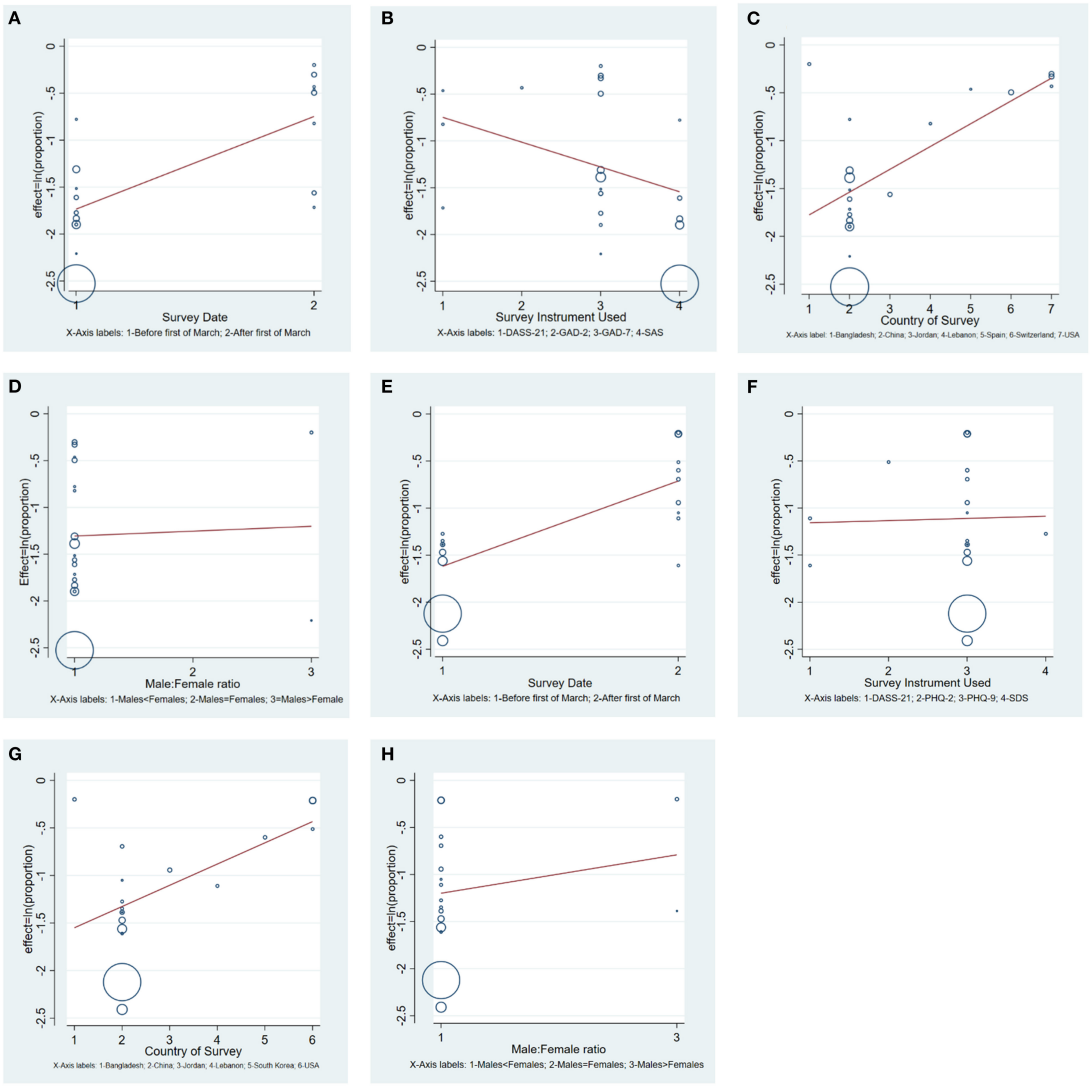
Meta-regression of pooled proportions (proportion = prevalence/100%) of anxiety and depression. **(A)** Anxiety proportion stratified by survey date; **(B)** anxiety proportion stratified by survey instrument; **(C)** anxiety proportion stratified by survey country; **(D)** anxiety proportion stratified by male-female ratio; **(E)** depression proportion stratified by survey dates; **(F)** depression proportion stratified by survey instrument; **(G)** depression proportion stratified by survey country; and **(H)** depression proportion stratified by male-female ratio.

Figures 1A–D summarizes the meta-regression of pooled proportions (Proportion = prevalence/100%) of anxiety stratified by survey date, survey instrument, survey country, and male-female ratio. The prevalence of anxiety was statistically significant higher in studies conducted after March the first [beta = 2.68; standard error (SE) = 0.67, *t* = 3.95, *p* = 0.001; CI: 1.58–4.55]. There was no statistically significant difference in the prevalence among studies using different survey instruments (i.e., DASS, GAD-2, GAD-7, or SAS) to assess anxiety [beta = 0.77; SE = 0.12, *t* = −1.64, *p* = 0.119; CI: 0.55–1.08].

Moreover, the prevalence of anxiety in Asian (i.e., Bangladesh, China, and Jordan) countries were lower as compared to European (i.e., Spain, Switzerland) and America [beta = 1.27; SE = 0.08, *t* = 3.86, *p* = 0.001; CI: 1.11–1.44]. Interestingly, studies with more males than females (i.e., male: female >1) had higher prevalence of anxiety than studies with more females than males (i.e., male; females <1) [beta = 0.26; SE = 0.09, *t* = 3.69, *p* = 0.002; CI: 0.12–0.56].

Having identified the confounders, we, therefore, pooled the overall prevalence of anxiety only including studies (Jiang et al., 2020; Liu et al., 2020; Wang and Zhao, 2020) conducted in the same country (i.e., China), on the same survey dates (i.e., before the first of March), and having the same male-female ratio (i.e., 0.7). The overall proportion was 0.15 [CI: 0.14–0.16] (i.e., prevalence = 15%) with non-significant heterogeneity (*I*^2^) of 63.8%, *p* = 0.063. There was no sufficient data to conduct this analysis in other countries.

## Prevalence of Depression

Figures 1E–H illustrates the meta-regression of pooled prevalence of depression stratified by survey date, survey instrument, survey country, and male-female ratio. Studies conducted after the first of March had a higher pooled prevalence of depression than before [beta = 2.48; SE = 0.52, *t* = 4.31, *p* = 0.001; CI: 1.58–3.87]. There was no statistically significantly difference in depression prevalence among different studies that used separate survey instruments [beta = 1.02; SE = 0.22, *t* = 0.11, *p* = 0.916; CI: 0.65–1.62].

Studies conducted in Asia reported a statistically significantly lower prevalence of depression than studies conducted in America and South-East Asia [beta = 1.25; SE = 0.096, *t* = 2.90, *p* = 0.01; CI: 1.06–1.47]. Again, studies with more males than females had higher prevalence of depression as compared to more females than males [beta = 0.25; SE = 0.082, *t* = 4.19, *p* = 0.001; CI: 0.12–0.50].

We pooled the overall prevalence of depression only including studies (Chang et al., 2020; Chi et al., 2020) conducted in the same country (i.e., China), on the same survey dates (i.e., before the first of March), having the same male-female ratio (i.e., 0.6) and used same survey instrument (i.e., PHQ-9). The overall proportion was 0.22 [CI: 0.20–0.24] (i.e., prevalence = 22%) with non-significant heterogeneity (*I*^2^) of 66.80%, *p* = 0.083. Other countries had no sufficient data to conduct this analysis.

## Conclusions

The differences in the college students' prevalence of depression and anxiety among nations and study survey dates, reported by Li et al., are statistically significant (i.e., *p* < 0.05). However, the prevalence of anxiety and depression are 15 and 22%, respectively, instead of 36 and 39% reported by Li et al. In addition, studies with more males than females had a statistically significant higher prevalence of anxiety and depression than studies with more females than males.

## Author Contributions

All authors listed have made a substantial, direct and intellectual contribution to the work, and approved it for publication.

## Conflict of Interest

The authors declare that the research was conducted in the absence of any commercial or financial relationships that could be construed as a potential conflict of interest.

## Publisher's Note

All claims expressed in this article are solely those of the authors and do not necessarily represent those of their affiliated organizations, or those of the publisher, the editors and the reviewers. Any product that may be evaluated in this article, or claim that may be made by its manufacturer, is not guaranteed or endorsed by the publisher.
